# Preliminary study on SPECT/CT imaging of pancreatic cancer xenografts by targeting integrin α5 in pancreatic stellate cells

**DOI:** 10.7150/jca.51190

**Published:** 2021-01-18

**Authors:** Tao Wang, Ye Peng, Rou Li, Xiao Li, Changjing Zuo

**Affiliations:** Department of Nuclear Medicine, Changhai Hospital, Naval Medical University, Shanghai 200433, China.

**Keywords:** pancreatic cancer, SPECT imaging, pancreatic stellate cells, integrin α5, targeting

## Abstract

**Background:** Integrin α5 (ITGA5) is overexpressed specifically in pancreatic cancer stroma, specially, in the activated pancreatic stellate cells (PSCs). Molecular imaging of pancreatic cancer via targeting PSCs has its advantages.

**Purpose:** This study aims to investigate the feasibility of ITGA5-targeted SPECT/CT imaging of pancreatic cancer by targeting PSCs.

**Methods:** ITGA5 expression in PSCs treated without or with pancreatic cancer SW1990 cells conditioned medium (SW1990-CM) was assessed by western blotting and immunofluorescence staining. ITGA5 specific inhibitor AV3 peptide was radiolabeled with ^125^I to synthesize ^125^I-AV3, and the labeling rate, *in vitro* stability and cellular uptake were further investigated. SW1990 cells alone or with PSCs were injected subcutaneously on the left and right lower limbs of nude mice respectively to establish pancreatic cancer xenograft model, and then ^125^I-AV3 SPECT/CT imaging of pancreatic cancer-bearing nude mice was performed. The expression of ITGA5 in tumors was detected by immunohistochemical (IHC) staining.

**Results:** ^125^I-AV3 has an excellent labeling rate and good *in vitro* stability. After treated with SW1990-CM, PSCs had an increased expression of ITGA5 and higher ^125^I-AV3 uptake. SPECT/CT imaging study showed that ^125^I-AV3 was mainly accumulated in the right xenografts (co-injection of cancer cells and PSCs), while the left xenografts tumors have a poor imaging. Moreover, the uptake of radiotracer in both side tumors was inhibited significantly after the non-radiolabeled AV3 pretreatment. IHC staining showed that SW1990 + PSCs tumor has a higher positive rate of ITGA5 than SW1990 tumor.

**Conclusion:** The preliminary study suggests that ^125^I-AV3 can be used for SPECT/CT imaging of pancreatic cancer via targeting ITGA5 in PSCs, which is independent of the state of cancer cells and may have a special meaning.

## Introduction

Pancreatic cancer remains one of the most aggressive and the least curable cancers, whose mortality is approximately equal to incidence. Nuclear medicine molecular imaging techniques such as single photo emission computed tomography (SPECT) and positron emission tomography (PET), which are quite different from the anatomical imaging including ultrasonography, CT and MRI, can provide the functional information based on the metabolic characteristics or specific biomarker of tumor, and have been widely used in preclinical or clinical diagnosis and management of pancreatic cancer 1. Specially, the clinical used ^18^F-FDG PET/CT and PET/MR have showed excellent performances for the initial staging, treatment plan determination, therapy response and prognosis evaluation of pancreatic cancer, and contribute to improve the tumor outcome 2-4. However, ^18^F-FDG PET/CT still has several drawbacks in detecting pancreatic cancer. The chronic and acute pancreatitis both have high ^18^F-FDG uptake, leading to false-positive interpretations, and the application of ^18^F-FDG PET in hyperglycemic patients also is not ideal, because the uptake of imaging agent is suppressed as a result of high serum glucose competitive inhibition 5,6. Therefore, the development of new molecular probes, which can target pancreatic cancer specifically and overcome the limitations of ^18^F-FDG to some extent, are urgently needed and have great clinical significance. For example, integrin α_v_β_6_ is specifically overexpressed in pancreatic cancer, and its targeting peptide has been radiolabeled with ^99m^Tc or ^68^Ga for the SPECT or PET imaging of pancreatic cancer respectively 7,8.

In human pancreatic cancer, the tumor stroma comprised of CAFs, inflammatory cells, blood vessels and extracellular matrix (ECM), can occupy up 90% of the entire tumor mass. PSCs, which are in a quiescent state in the healthy pancreas, but become activation under various kinds of stimuli including inflammatory and carcinogenic processes, are considered as the main source for CAFs and the most prominent cell type of pancreatic cancer stroma 9. A recent study suggests that ITGA5 is overexpressed specifically in the tumor stroma of pancreatic cancer (mainly in CAFs or the activated PSCs), but has a low to negligible expression in cancer cells or normal pancreas 10. The overexpression of ITGA5 makes it a promising target for pancreatic cancer imaging by targeting tumor stroma, CAFs or activated PSCs. Considering that the high clinical heterogeneity of cancer cells, CAFs-targeted imaging of various cancers including pancreatic cancer has attracted special interest, especially the emergence of ^68^Ga-FAPI, which targets the roots of the tumor microenvironment rather than the cancer cells themselves and has a high potential to avoid some limitation existed in ^18^F-FDG 11,12.

In our preliminary study, we try to investigate the feasibility of SPECT/CT imaging of pancreatic cancer by targeting ITGA5 in PSCs using ^125^I-labeled ITGA5 inhibitor AV3 peptide as the imaging agent. The pancreatic cancer xenografts animal model was established through the con-injection of cancer cells and PSCs, which is more consistent with the clinical pathological characteristics of human pancreatic cancer. This study offers a possibility for pancreatic cancer imaging research via targeting the stroma cell (e.g. PSCs), which is independent on the status of cancer cells and helpful to overcome the heterogeneity of cancer cells.

## Methods

### Materials and regents

The human pancreatic cancer SW1990 cells and PSCs were obtained from Tongpai (Shanghai) Biotechnology Co., LTD. Anti-ITGA5 antibody and FITC-labeled secondary antibody were purchased from Beijing Bioss Biotechnology Co., LTD. DMEM and fetal bovine serum (FBS) were purchased from Gibco Life Technology Co., LTD. The AV3 peptide (purity = 98.86%) were synthesized by ChinaPeptide Co., Ltd. Na^125^I solution was obtained from Shanghai Xinke Pharmaceutical Company. Iodogen tubes pre-coated with iodogen were purchased from Shanghai Nice-labeling Biotech Co., Ltd. Other common regents were commercially available.

### Cell culture and cell experiments

SW1990 cells and PSCs were cultured in DMEM containing 10% FBS at 37 °C in a 5% CO_2_ incubator. For the preparation of SW1990-CM, SW1990 cells in a 10-cm dish were washed twice with PBS and then cultured with DMEM (10 mL) without FBS for 48 h. Then, the supernatant was centrifuged for 10 min at 3000 rpm at 4 °C, filtrated with 0.22 μm filters and eventually collected as SW1990-CM. To observe the influence of the tumor microenvironment on the ITGA5 expression of PSCs, when PSCs reached about 80% confluence, the medium was replaced by DMEM (as control group) or SW1990-CM and continue to culture for 12 h, and then ITGA5 was detected by western blotting and cell immunofluorescence staining.

For western blotting, PSCs were washed twice with PBS and then lysed using RIPA lysis buffer. After the measurement of the protein concentration using BCA assay, the protein lysates were separated by 10% SDS-PAGE and then transferred to a PVDF membrane. The membrane was blocked with 5% non-fat milk for 1 h in TBST at room temperature. After this, following the recommended concentration, the PVDF membranes were incubated with anti-ITGA5 antibody or GAPDH monoclonal antibody overnight at 4 °C, followed by HRP-conjugated secondary antibody at room temperature for 1 h. Lastly, the membrane was rinsed twice with TBST, and the bands of membrane were visualized using Tanon 5200 chemiluminescence detection system. The relative density of target bands, which represents the expression level of ITGA5, was quantified using Image J software. The immunofluorescence staining experiment was performed following our previous description 13.

### Synthesis and *in vitro* stability test of ^125^I-AV3

AV3 peptide (RYYRITY), which contains three tyrosine residues, meaning it is very suitable for radioiodine labeling, was radiolabeled with ^125^I using the Iodogen method similar with our previous reported procedures 13. The mixture of 20 μg AV3 peptide (dissolved in 200 μL water) and 2 mCi Na^125^I were added into an Iodogen tube containing 50 μg Iodogen, and about 10 min later, the reaction was ended by separating the supernatant (^125^I-AV3) and iodogen. The labeling rate of ^125^I-AV3 was measured using a mini-scan radio thin layer chromatography (TLC) scanner where Xinhua No.1 chromatography filter paper and water were employed as the stationary phase and the mobile phase respectively. The labeling rate can be calculated according to the quantification result of radioactive bands with the help of the system software. For the *in vitro* stability test, ^125^I-AV3 was mixed with PBS or 10% FBS firstly, and then at different time points (0.5, 1, 4, 8, 12, 24 h), the radiochemical purity (RCP) of ^125^I-AV3 solution in PBS or 10% FBS were assessed following the above-described TLC method.

### Cellular uptake of ^125^I-AV3

The cellular uptake difference of ^125^I-AV3 between PSCs and SW1990-CM-treated PSCs was compared. PSCs or SW1990-CM-treated PSCs were seeded in a 6-well plate at a density of 2 × 10^5^ cells/well, and about 50 μCi ^125^I-AV3 was added into each well. Cells were further cultured at 4 °C for 0.5 h, 2 h or 4 h, and at each time point the free ^125^I-AV3 was removed by washing cells twice with PBS. Then, 0.1 M NaOH was added to solubilize the cells, and the cell-bound radioactivity (counts per minute, CPM) of well was measured using a γ-counter.

### Establishment of pancreatic cancer xenograft model, SPECT/CT imaging and ITGA5 IHC staining

Six-week-old nude mice were provided by the Laboratory Animal Center of Naval Medical University, and all animal procedures were conducted in accordance with the institutional guidelines. To establish pancreatic cancer-bearing animal model, 100 μL SW1990 cells suspension (1 × 10^6^ cells) alone or with PSCs (5 × 10^6^ cells) were subcutaneously injected into the left and right lower limbs of nude mice respectively. The tumors were observed and when the diameters of tumors exceeded 1.0 cm, the imaging study began. The nude mice were anesthetized and received an injection of 1 mCi ^125^I-AV3 by tail vein. SPECT/CT imaging of pancreatic cancer-bearing nude mice was typically performed at 4 h after injection using a clinical used SPECT/CT scanner (Symbia T16, Siemens, Germany). For the *in vivo* blocking experiment, excessive non-radiolabeled AV3 (about 100 times) was injected prior to the injection of ^125^I-AV3, and then SPECT/CT imaging was carried out. The co-registration of SPECT and CT images were performed and the fusion images were quite visually intuitive to assess the radiotracer uptake in tumors.

After imaging, pancreatic cancer-bearing nude mice were executed with overdose anesthesia and the tumors in both lower limbs were cut off, and then ITGA5 expression in tumors was assessed by IHC staining in accordance with the corresponding protocols.

### Statistical analysis

Quantitative data are expressed as the means ± SD, and the difference between two groups was compared by student *t*-test using GraphPad Prism 5 software. *P* < 0.05 was considered statistically significant.

## Results

### SW1990-CM promoted the expression of ITGA5 in PSCs

**Figure 1:**
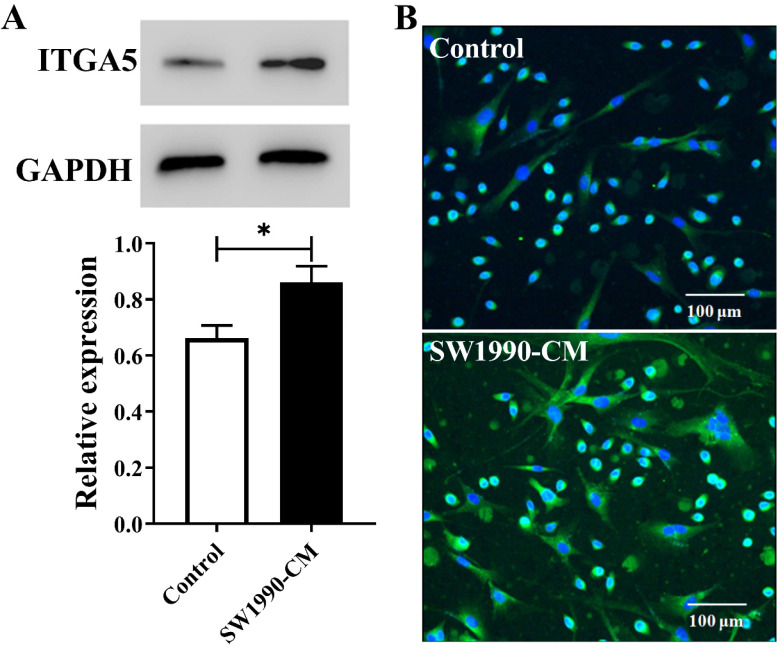
Western blotting assay (A) and cell immunofluorescence staining (B) of ITGA5 expression in PSCs without or with SW1990-CM treatment. *P < 0.05, n = 3.

Inside pancreatic cancer, the tumor microenvironment induces PSCs activation, and the activated PSCs are the major contributor to the malignant biological behavior of pancreatic cancer. Cancer cell conditioned medium often is chosen to mimic this microenvironment. In present study, according to the results from Figure 1, after the treatment with SW1990-CM, ITGA5 expression in PSCs has a significant increase, meaning that PSCs activation occurred and our used pancreatic cancer xenografts, which were established by the co-injection of cancer cells and PSCs, would have a high expression of ITGA5 in the tumor stroma or the activated PSCs.

### The *in vitro* stability and cellular uptake of ^125^I-AV3

**Figure 2:**
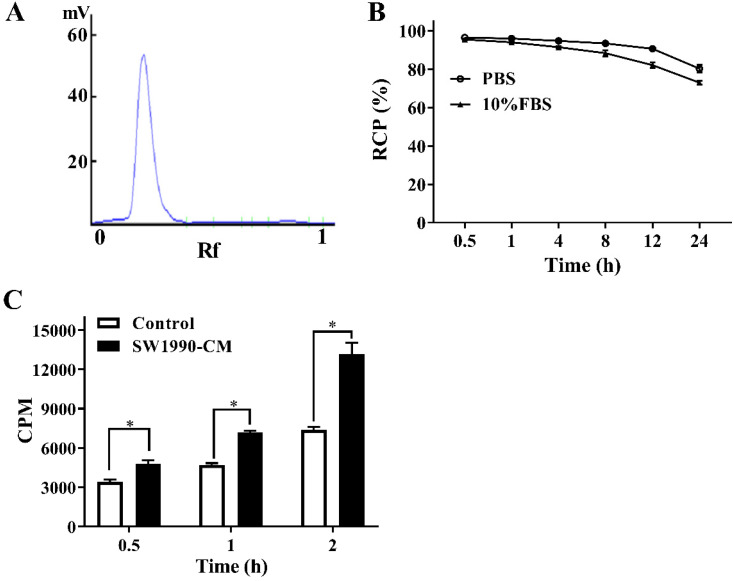
The labeling rate (A), *in vitro* stability (B) and cellular uptake (C) of ^125^I-AV3. *P < 0.05, n = 3.

An excellent labeling rate (over 98%) of ^125^I-AV3 makes it can be used directly without further purification (Figure 2A). As for the* in vitro* stability, at the first 4 h, the de-iodination of the radiotracer was very slowly, and up to 12 h after incubation, the radiochemical purity of ^125^I-AV3 still reached 91%, 82% in PBS and 10% FBS, respectively, suggesting a not outstanding but still good *in vitro* stability (Figure 2B). As shown in Figure 2C, the ^125^I-AV3 uptake in the quiescent and activated PSCs both increased over time, but at each time point, the CPM in SW1990-CM-treated group was higher than that in control group, which can be attributed to a higher expression of ITGA5 in the activated PSCs.

### SPECT/CT imaging of pancreatic cancer by targeting PSCs

**Figure 3:**
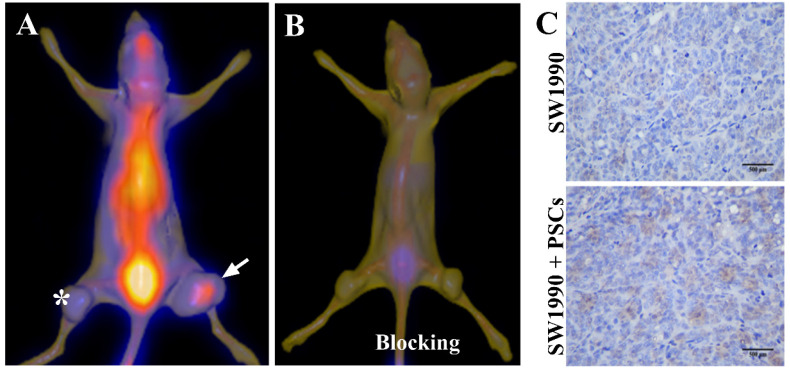
Representative ^125^I-AV3 SPECT/CT imaging at 4 h (A), corresponding SPECT/CT imaging with a blocking dose of AV3 (B) and ITGA5 IHC staining (C) of pancreatic cancer xenografts in nude mice. The xenografts tumors in nude mice were established using SW1990 cells injection alone in the left lower limb (indicated by asterisk) or SW1990 + PSCs co-injection in the right lower limb (indicated by arrow). Pancreatic cancer-bearing nude mice were injected about 1 mCi ^125^I-AV3 from tail vein before the imaging study. Repeated experiments showed similar results.

Given that the activated PSCs play a vital role in the development and progress of pancreatic cancer, co-injection of SW1990 cells and PSCs was adopted to establish the pancreatic cancer xenografts model in our study, and the formed tumors have bigger size compared with SW1990 cells injection alone (Figure 3A). ^125^I-AV3 SPECT/CT imaging of pancreatic cancer-bearing nude mice showed that the radiotracer was mainly accumulated in the right tumor, and the xenografts tumor in the left lower limb has a very weak imaging, suggesting that ^125^I-AV3 can target the ITGA5-expressing PSCs rather than cancer cells (Figure 3A). In blocking experiment, the uptake of radiotracer in both side tumors was inhibited significantly due to the pretreatment with excessive non-radiolabeled AV3 peptide, indicating that the binding of ^125^I-AV3 and ITGA5 is specific *in vivo* (Figure 3B). In addition, IHC staining further confirmed the tumor status, in which SW1990 + PSCs tumor has a higher positive rate of ITGA5 than SW1990 tumor (Figure 3C).

## Discussion

The activated PSCs constitute over 50% of the tumor stroma in pancreatic cancer, and also are the most important stroma cell type and the main source of CAFs 9. In the field of nuclear medicine molecular imaging, the targeted SPECT and PET imaging of pancreatic cancer is often based on the specific biomarkers expressed in cancer cells (e.g. integrin α_v_β_6,_ tissue factor, neurotensin receptor 1) or neo-vascularendothelial cells (e.g. integrin α_v_β_3_), but few researches focus on the CAFs or PSCs 8,14-16. However, given that cancer cells constitute a minority but with a high heterogeneity in pancreatic cancer, targeting CAFs or the activated PSCs has its unique advantages and has become one of the research hotspots 17,18.

ITGA5 is an interesting integrin receptor, which is overexpressed specifically in pancreatic cancer stroma and, further, CAFs or the activated PSCs 10. Consistent with previous reports that cancer cells-conditioned medium induce PSCs activation and enhance the expression of ITGA5 in PSCs 10,19, our *in vitro* results showed that the expression of ITGA5 in PSCs increased because of the indirect effect of cancer cells, which is the foundation of SPECT imaging of pancreatic cancer. In our study, different from the most common strategy of targeting pancreatic cancer cells, CAFs or the activated PSCs-targeted SPECT/CT imaging of pancreatic cancer was achieved using the ITGA5-targeted molecular probe ^125^I-AV3. The pancreatic cancer xenograft model usually is established by the injection of cancer cells alone, but the important role of stroma cells (e.g. CAFs or PSCs) is grossly neglected, which is inconsistent with human tumor and also is one of the reasons that many laboratory results fail to translate for clinical practice. Our used pancreatic cancer animal model, which was established via cancer cells injection alone and the co-injection of cancer cells and PSCs in the same nude mouse, not only reflected the pro-tumorigenic effect of PSCs, but also confirmed that the existence of PSCs made the xenograft tumor has a higher ITGA5 expression and ITGA5-expressing PSCs rather than cancer cells had an effective uptake of ^125^I-AV3, suggesting the feasible of pancreatic cancer imaging by targeting CAFs or the activated PSCs.

As a preliminary study, the feasibility of ^125^I-AV3 SPECT/CT imaging of pancreatic cancer by targeting ITGA5-expressing PSCs has been confirmed, but there are still some shortcomings. For example, the radioiodine labeling of AV3 peptide is very simple and efficient, but ^125^I itself is an imperfect SPECT radionuclide compared with other radionuclides such as ^99m^Tc, together with the application of the clinically used SPECT/CT system rather than the small animal micro-SPECT/CT, resulting in difficult to assess the tissue and organ distributions of ^125^I-AV3 from the low resolution SPECT/CT images. In addition, the *in vivo* stability of ^125^I-AV3 seems to be unsatisfactory. The selective binding, uptake and stability of linear peptide can be enhanced by cyclization 20, and a cyclic AV3 peptide may be a better choose. Based on the preliminary study, ^68^Ga labeled cyclic AV3 for micro-PET/CT imaging of pancreatic cancer-bearing nude mice is worth investing in further.

In conclusion, our preliminary study suggests that^ 125^I-AV3 can be used for SPECT/CT imaging of pancreatic cancer via targeting ITGA5 in PSCs, which is independent of the state of cancer cells and may have a special meaning.
